# The Humoral Immune Response to HCV: Understanding is Key to Vaccine Development

**DOI:** 10.3389/fimmu.2014.00550

**Published:** 2014-11-10

**Authors:** Siobhán B. Cashman, Brian D. Marsden, Lynn B. Dustin

**Affiliations:** ^1^Nuffield Department of Orthopaedics, Rheumatology, and Musculoskeletal Sciences, Kennedy Institute of Rheumatology, University of Oxford, Oxford, UK; ^2^Nuffield Department of Medicine, Structural Genomics Consortium, University of Oxford, Oxford, UK

**Keywords:** HCV, HCV/E2 glycoprotein, monoclonal antibodies, viral antibodies, chronic infection, acute infection, neutralizing antibodies

## Abstract

Hepatitis C virus (HCV) remains a global problem, despite advances in treatment. The low cost and high benefit of vaccines have made them the backbone of modern public health strategies, and the fight against HCV will not be won without an effective vaccine. Achievement of this goal will benefit from a robust understanding of virus–host interactions and protective immunity in HCV infection. In this review, we summarize recent findings on HCV-specific antibody responses associated with chronic and spontaneously resolving human infection. In addition, we discuss specific epitopes within HCV’s envelope glycoproteins that are targeted by neutralizing antibodies. Understanding what prompts or prevents a successful immune response leading to viral clearance or persistence is essential to designing a successful vaccine.

## Introduction

Between 130 and 185 million people worldwide are infected with hepatitis C virus (HCV) and are at risk of cirrhosis, hepatocellular carcinoma, and end-stage liver disease (1–3). HCV, a member of the *Flaviviridae* family, is parenterally transmitted. HCV establishes a persistent infection in 60–80% of individuals infected. The treatment for HCV has long been pegylated interferon alpha co-administrated with ribavirin, but the response rates were unsatisfactory with only 50–60% of patients achieving a sustained virologic response (4, 5). The welcomed discovery of new directly acting antiviral drugs (DAAs) is expected to lead to a dramatic increase in cure rates (6–8). However, it is unlikely that the global HCV problem will be eliminated any time soon. There are numerous challenges that must be overcome first, including the prohibitive cost of treatment and the need for new treatment strategies for patients with advanced liver disease or co-morbidities (9). Another important obstacle is identifying those in need of treatment, since symptoms may be absent or non-specific until after significant liver damage has set in (10).

The development of a protective vaccine is essential in combating the global HCV epidemic. Understanding the immune response in those who spontaneously resolve HCV infections versus those who develop chronic infection is key to the development of prophylactic or therapeutic vaccine (11). So far, developing a HCV vaccine has proven challenging, not least because HCV is genetically highly diverse; there are seven known major genotypes that differ from each other by 30–35%, and over 60 subtypes (12). Indeed, the virus exists as a quasispecies – a swarm of related but distinct sequences – within an infected patient. This diversity is a consequence of HCV’s high replication rate, and an RNA polymerase that lacks any proofreading mechanism. High viral diversity within and between infected individuals poses challenges to vaccine developers: how can we devise a vaccine that will stimulate broadly cross-reactive immune responses to such a changeable foe? The key may well be to target an array of viral epitopes that are functionally constrained, and to enlist both humoral and cellular arms of the adaptive immune response. In particular, it will be important for the vaccine to elicit neutralizing antibodies (nAbs) to block viral access to target cells, and T-cell responses targeting infected cells (13).

Adaptive immune responses are typically delayed during acute HCV infection. HCV RNA can be detected 1–3 weeks following infection, but neither HCV-specific T-cells nor HCV-specific antibodies (Ab) are observed until 1–2 months after infection (14–18). Both CD4^+^ and CD8^+^ T-cell responses play essential roles in the outcome of infection. CD8^+^ T-cells limit HCV replication through cytolytic and non-cytolytic immune mechanisms that are highly dependent on CD4^+^ T-cell function [reviewed in Ref. (19–23)]. Vigorous and broadly directed anti-HCV T-cell responses are observed in patients who resolve infection (24–27). In patients who progress to chronicity, initial vigorous T-cell responses wane and weaken. Loss of CD4^+^ T-cell help, a switch to a T_reg_ cell profile, viral epitope escape, and chronic antigen stimulation may all contribute to T-cell exhaustion (23).

It was widely thought that the humoral immune response to HCV played only a peripheral role in HCV infection (24, 28, 29). However, recent studies suggest that B-cells and nAbs may play active roles in the spontaneous resolution of HCV (30–33). Typically, an nAb response would be a component of sterilizing antiviral immunity and has long been a quintessential part of vaccine design (13, 34). An HCV vaccine will need to stimulate strong humoral as well as cellular immune responses. The role of humoral immune system in the both the control of HCV infection and in the pathogenesis of liver disease is still unclear. In this review, we hope to outline our current understanding of the humoral immune system’s roles in acute infection, the progression to chronicity, and the spontaneous resolution of HCV infection, and to highlight some of the pressing questions that need to be addressed.

## nAb Epitopes

Antibodies produced during acute HCV infection target epitopes within both structural and non-structural (NS) viral proteins. However, all known nAbs target epitopes within the HCV envelope glycoproteins E1 and E2, or the E1E2 heterodimer. The structural proteins core, E1, and E2 are released from the viral polyprotein by cellular signal peptidases. The viral particle contains the nucleocapsid, formed by the close interaction of the HCV RNA genome and core protein, surrounded by a lipid bilayer envelope into which the glycoproteins E1 and E2 are anchored. E1 and E2 form a heterodimer that mediates viral entry. Determining the structure of the E1E2 heterodimer has proven problematic. E2 is required for the correct folding of E1, so that E1’s structure is still uncertain (35, 36). It is thought that the glycans on both the heavily glycosylated E1 and E2 are involved in folding of the E1E2 heterodimer (37). Interestingly, nAbs (AR4 and AR5) have been found that recognize conformational epitopes on the E1E2 heterodimer with broad neutralizing crossreactivity between diverse HCV genotypes (32).

Most nAbs target E2. E2 plays a key role in HCV entry, directly interacting with two of the cellular proteins needed for viral entry, CD81 and scavenger receptor class B type I (SR-BI) (38, 39). CD81 and SB-RI alone are not sufficient for viral entry; tight junction proteins claudin-1 and occludin are also required (40, 41). Other factors, such as the cholesterol absorption receptor Niemann-Pick C1-like 1, epidermal growth factor receptor, ephrin receptor type A2, and most recently, transferrin receptor 1 enhance viral entry (42–44).

**Figure 1:**
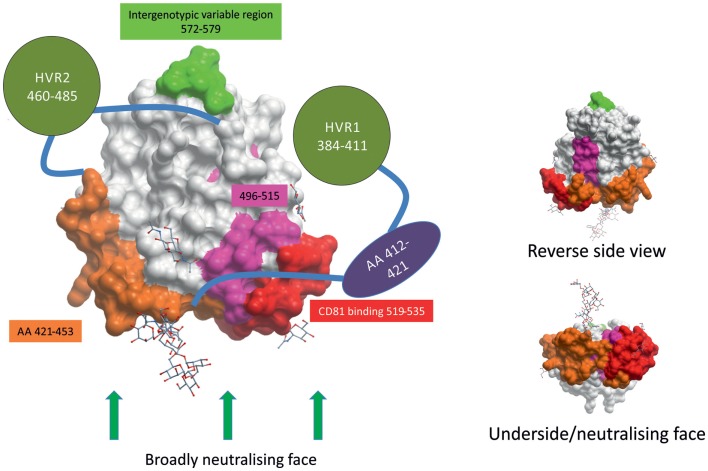
**HCV envelope glycoprotein 2 surface representation**. E2 is a globular protein with three regions of hypervariablity – HVR1, HVR2, and intergenotypic variable region – shown in green. Domains whose structures are currently unknown are depicted as shapes apart from the structure. HVR1 is predicted to mask a hydrophobic region that is sensitive to nAbs. E2’s broadly neutralizing face, where many broadly neutralizing Abs bind, comprises CD81-binding loop (in red), residues 421–453 (in orange), residues 502–520 (in pink), and residues 412–421 (in purple). The possible positions of some glycans are shown as stick and ball figures. E2 structure obtained from PDB (4MWF) (45).

Two recent reports have shed light on the structure of E2 (45, 46). Most surprisingly, E2 did not adopt the expected, highly extended conformation of class II fusion envelope protein like other members of *Flaviviridae*, such as Tick-borne encephalitis virus or West Nile virus; instead, HCV E2 was found to be compact and globular (Figure 1), with a central beta sandwich surrounded front and back by short alpha helices, loops, beta sheets, and regions lacking organized structure (45, 46).

The ectodomain of E2 contains three regions of variability that are targeted by nAbs: hyper-variable region (HVR) 1, HVR2, and intergenotypic variable region. HVR1 contains dominant neutralizing epitopes, and its variation leads to immunological escape (47–50). HVR1 is a 26–28 amino acid segment located near the amino terminus of E2. HVR1 interacts with SR-BI and is, therefore, likely involved in virus entry, making it an interesting target for nAbs (51, 52). The most-effective nAbs that target HVR1 recognize epitopes found in the C-terminus, where HVR1 interacts with SR-BI (52–54). However, HVR1 mutates rapidly and antibodies that recognize HVR1 demonstrate very poor cross-neutralization across different isolates of the same HCV genotype (50, 55, 56). As of yet, no HVR1 nAbs have been found that display broadly cross-reactive neutralizing activity.

CD81 was the first host receptor identified as being a crucial entry factor for HCV and, therefore, the CD81-binding region of E2 is a likely target for nAbs (38). Indeed, numerous broadly nAbs have been found to target the CD81-binding loop (residues 519–535, numbered according to the HCV H77 consensus sequence accession number AF009606) of E2 (30, 32, 45, 57–60). Kong and colleagues found evidence to suggest CD81 interacts with both the CD81-binding loop (519–535) and residues 421–453 of E2, making this domain in E2 a most intriguing target for vaccine design (Figure 1) (45). nAbs have also been found that target the less variable region directly downstream of HVR1 (residues 412–421) (61–63). However, less than 5% of spontaneous resolvers have nAbs targeting this region, suggesting that this region is not as immunogenic *in vivo* (64). A highly conserved neutralizing epitope encompassing residues 496–515 is located between the two CD81-binding regions of E2 (Figure 1). Although not directly involved in CD81 binding, binding of nAbs to the 496–515 epitope may cause a conformational change in the CD81-binding region (65). Contrary to what was previously thought, the overlapping residues 502–520 do not contain a fusion peptide (66, 67).

Most of the identified nAbs target regions within E2 that interact with the viral entry factors CD81 and SR-BI. Occludin is also thought to interact directly with E2 (68); perhaps mapping of this interaction will allow the development of novel nAbs. In contrast, claudin-1 has not been shown to interact directly with E2. Would targeting the viral epitope that recognizes claudin-1 prevent entry? Perhaps we know that blocking claudin-1 prevents E2/CD81/claudin-1 interaction, thereby preventing HCV entry (69). Targeting host factors such as SR-BI, CD81, occludin, or claudin-1 may provide novel therapy options that could be used in conjunction with current treatments (70).

Few nAbs have been identified that specifically target E1 (11, 71). Whether this is due to the poor immunogenicity of E1 is still unclear. Anti-E1 Abs are only detected at low levels in HCV patients (72, 73). A recent study reported that patients develop almost sevenfold more antibodies to E2 than to E1 (study in four patients); however, the E1-specific Abs contributed largely to the overall neutralization of HCVpp, despite the relatively low number of E1-specific Abs compared to E2 (74). Anti-E1 Abs have proven difficult to induce in vaccines expressing the E1E2 heterodimer, and are more efficiently induced by expressing E1 alone (75). Whether this is due to the immunodominance of E2 or the masking of E1 neutralizing epitopes by E2 is unknown. Two broadly neutralizing epitopes in E1 encompass residues 192–202 (76) and 264–327 (65, 74, 77). Little is known about the structure, function, and interactions of E1. We know that E1 is involved in E2 folding (78). It is uncertain how E1-specific nAbs mediate their activity. It has been suggested that E1 may contain the element needed for envelope membrane fusion; as such E1 and the (as yet unidentified) fusion epitope it may contain remain an intriguing target for nAbs (79).

The HCV virion forms a complex with low-density and very low-density lipoproteins, forming a lipoviral particle (80). Studies of lipoviral particles showed that host lipoprotein apoE was incorporated into particles with significantly more apoE incorporated than E2 (81, 82). Lipoproteins limit the amount E1E2 to be seen on the surface of the lipoviral particle (82).

Interestingly, many HCV diagnostic assays detect Abs directed against both structural and NS HCV proteins. Why are NS proteins targeted? There is little evidence to suggest they are incorporated into the viral particle, yet NS3-specific Abs are detected before envelope-specific Abs during acute HCV infection (17). It is not yet clear when and in what form the NS proteins are exposed to B-cells. Perhaps NS protein-specific Abs are produced in response to debris from damaged cells. Abs binding to non-neutralizing targets may aid in clearance through opsonization.

## Systems for Studying Abs

The study of Ab responses in HCV infection was long hampered by the lack of a cell culture system or permissive small animal model. Early *in vivo* studies, conducted in chimpanzees, confirmed the presence of nAbs in plasma from a human being with chronic HCV infection (83). Initial *in vitro* research on the effect of nAbs in HCV exploited E2/CD81 interaction to perform a neutralization of binding assay with recombinant E2 (38). This system was limited, as it could only evaluate putative neutralizing epitopes overlapping with the E2/CD81-binding region, and likely underestimated the quantity and complexity of nAbs present. Virus-like particles (VLP), produced in a baculovirus system, expressed the E1E2 glycoproteins in a more native conformation (84). The development of retroviral pseudoparticles (HCVpp) expressing unmodified E1E2 glycoproteins that has permitted a more in-depth study of HCV-specific nAbs (50, 85). The HCVpp system is adaptable to allow the expression of E1E2 glycoproteins from diverse HCV genotypes as well as the expression of patient-derived E1E2 (86). This allows patient sera to be screened for neutralizing activity against autologous viral envelope glycoproteins. E1E2 sequences may be cloned from patients’ serial samples, permitting the study of quasispecies and nAb co-evolution over time. Unfortunately, a disadvantage of this system is that the structure and neutralization requirements of HCVpp are still significantly different from those of authentic hepatocyte-derived HCV. The cell cultured derived HCV system (HCVcc) (87–90) may help overcome some of the limitations of the HCVpp system.

## Humoral Immune Response in Infection

While the majority of HCV-infected patients progress to chronic hepatitis with persistent viremia, a significant number (up to 40%) of patients spontaneously clear the infection depending on factors such as, race, sex, and genetics (91–95). It is widely accepted that the cellular immune response can mediate clearance of HCV infection [reviewed in Ref. (23)], but the role of the humoral immune response in acute infection and spontaneous clearance is not fully understood. nAbs are produced in response to HCV infection, but their contribution to control of infection is unclear (83). The acute humoral immune response to HCV has proved challenging to study, as most often patients are asymptomatic and unaware of their infection status; many studies have been retrospective. Typically, IgM is the first immunoglobulin isotype produced by the humoral system in response to infection; however, HCV-specific IgM has not proved a good marker of acute HCV infection as HCV-specific IgM is readily detected in chronically infected patients (96, 97), and HCV-specific IgM and IgG are both almost simultaneously detected in acute infection (98, 99).

There is much evidence to support the theory that Abs have a limited impact on HCV disease outcome as HCV seroconversion is delayed (15, 17, 100, 101), nAbs that target E1E2 are readily detected in the serum of chronically infected patients (29, 102–104), HCV-specific Ab titers wane in patients who have controlled the infection (17, 29, 105, 106), and there are numerous reports of the clearance of HCV infection in the absence of any detected HCV-specific Ab response (15, 28, 107–110).

In contrast, there is striking evidence supporting a role for Abs in control of HCV infection and more interestingly in preventing reinfection. Early induction of cross-reactive nAbs during acute infection strongly correlates with the spontaneous clearance of HCV (31, 33, 50, 73, 111, 112). Conversely, in patients who became persistently infected (>1 year), nAbs were delayed and initially had a narrow neutralizing range, which widened over time (31, 33, 73). In one remarkable case, a patient with established chronic HCV developed a broadly reactive nAb response followed by spontaneous viral clearance (111). Even in chronicity, nAbs may mediate some control of HCV infection as hypogammaglobulinemic patients experience a more rapid and severe progression of disease (113), and patients treated with rituximab show an increase in viral load, which returns to pretreatment levels after completion of treatment (114). Studies in cohorts of intravenous drug users have shown that individuals who spontaneously resolved one episode of HCV infection were more likely to clear a subsequent HCV infection (31, 91, 94, 115), and the time taken to clear the reinfection was significantly shorter (31, 115). Similar to observations in cohorts of intravenous drug users, >80% of chimpanzees that have previously spontaneously cleared HCV, rapidly clear a second infection (16, 109, 116), supporting the hope that protective immunity may be an achievable goal. More work is needed to elucidate the contribution of Abs to the clearance of in HCV in reinfection.

Passive immunization with nAbs can mediate protection: chimpanzees passively immunized with rabbit antisera specific for E1 and E2 were somewhat protected against HCV (117). Recently, HCV1, a human monoclonal Ab targeting E2, has been shown to prevent HCV infection and to reduce the viral load in chronically infected chimpanzees (118). Passive immunity is possible in human beings – it is best highlighted by the Gammagard incident (119). In the early 1990s, the makers of Gammagard (an immunoglobulin product prepared from pooled human plasma), in a move to improve the safety of their product, excluded sera containing anti-HCV Abs from the donor pool; unfortunately, the pooled preparations that removed the HCV seropositive serum lots transmitted HCV to patients (120, 121). Removing HCV-specific Abs from the product removed the protection provided by nAbs previously present. Subsequent screening of the product found HCV RNA (122). HCV-specific Abs provide immunity; however, it is not a sterilizing immunity, as demonstrated by the presence of high-titer nAbs in many patients with persistent HCV infection.

## Vaccine Trials

Vaccination has been the most-effective strategy used to control infections that have been a major public health concern. The hepatitis B virus (HBV) vaccine has proven a great success, greatly reducing the number of HBV infections worldwide (123). All successful viral vaccines that have been developed to date induce nAbs (13). However, an HCV vaccine has remained elusive. Most HCV vaccine trials have been conducted in chimpanzees, the best model permitting challenge with infectious HCV. A meta-analysis of HCV vaccine trials in chimpanzees has shown that the vaccines with greatest success contained part or all of the HCV envelope region inducing nAb responses, generating humoral or both humoral and cellular immune responses (124). Recently, a recombinant E1E2 vaccine (derived from HCV 1a) induced protective humoral immune responses in chimpanzees challenged with homologous or heterologous HCV 1a strains (125), and was approved for phase I clinical trial in human beings. In the clinical trial, the recombinant E1E2 vaccine induced antibody and cellular responses in healthy volunteers (126). Further investigation showed the vaccine-induced nAbs against heterologous HCV 1a strains in some healthy volunteers, and one volunteer (out of 16 tested) produced broadly cross-neutralizing Abs against all 7 HCV genotypes (127). A prophylactic vaccine that blocks all infection upon exposure would be ideal. However, preventing the progression of HCV infection to chronicity through a therapeutic vaccine may be a more realistic goal (124, 128, 129).

## Escape and Evasion of the Humoral Immune Response

**Figure 2:**
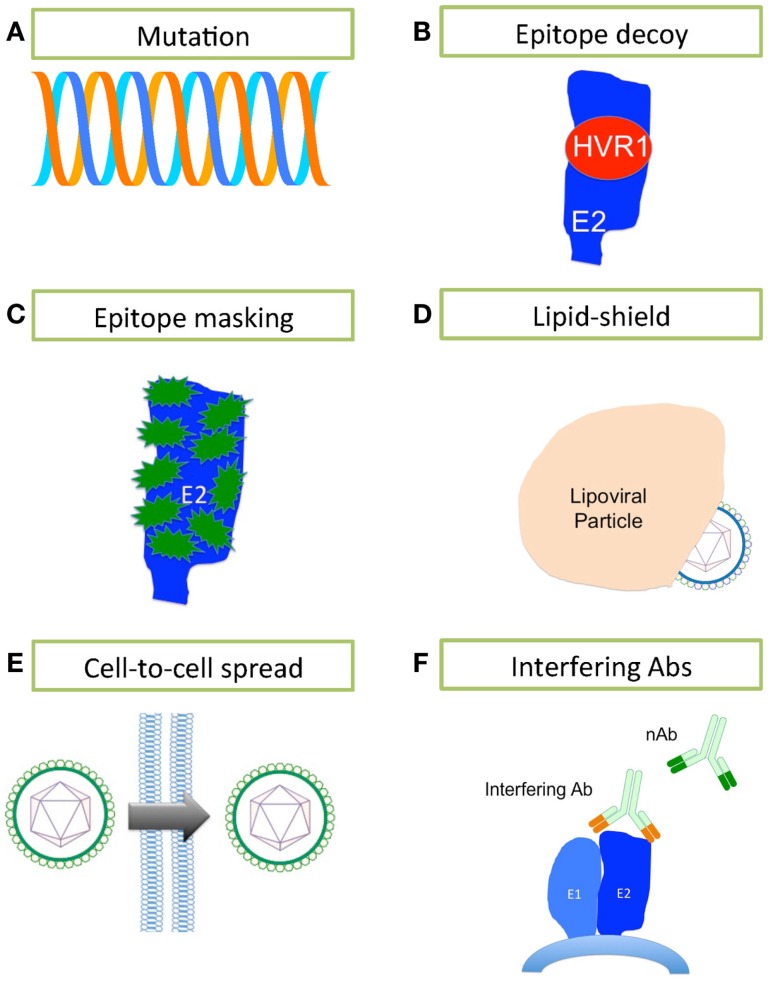
**Mechanisms of HCV evasion of the humoral immune system**. HCV has developed several strategies for evading the humoral immune system. **(A)** A high rate of replication using a polymerase that lacks any proofreading mechanism leads to generation of a rapidly changing quasispecies. **(B)** The highly immunogenic HVR1 masks a hydrophobic region that is sensitive to nAbs. **(C)** E2 is heavily glycosylated, containing between 9 and 11 N-linked glycans (in green) that mask much of the surface of E2 from nAbs. **(D)** The close association between HCV and lipoprotein effectively conceals the lipoviral particle from nAbs. **(E)** HCV may bypass the extracellular space and nAbs by spreading cell-to-cell. **(F)** Interfering antibodies can disrupt the action of nAbs by binding to non-neutralizing sites and masking neutralizing sites nearby.

Neutralizing antibodies are induced during HCV infection, which in some patients contribute to the spontaneous clearance of infection, yet the majority of infected patients progress to chronicity. How does HCV evade the humoral immune response to progress to chronicity? Several mechanisms may contribute to evasion of sterilizing Ab-mediated clearance. These include sequence changes, decoy epitopes, epitope masking, lipid shielding, induction of interfering antibodies, and the ability to move from one cell to another in a neutralization-resistant fashion (Figure 2).

Hepatitis C virus’ error-prone replication mechanism permits rapid escape from Ab-mediated and other pressures. Each day, an estimated 10^12^ new HCV virions are produced in the infected liver (130); it is estimated that thousands of virions bearing each possible single and double nucleotide substitution are made daily in an infected person (131). The resulting quasispecies swarm provides the raw material for selection of nAb-resistant populations (50, 132, 133). In fact, there are numerous reports that link viral sequence evolution, particularly within the E2 glycoprotein, to nAb escape in chronic infection (Figure 2A) (49, 50, 132, 133). Host nAb responses lag behind the rapidly mutating E2 sequences within the quasispecies (50, 132). That nAbs fail to neutralize the dominant viral strain at a given time, yet successfully neutralize previously dominant viral strains in the same patient, clearly demonstrates the continued evolution and escape of the virus under selective pressure from nAbs, with the humoral immune system always, alas, one step behind (132).

It has been suggested that HVR1 of E2 acts as an immunological decoy (Figure 2B) (71, 134). HVR1 is highly immunogenic, but is not essential for viral entry/infection (135); however, HVR1 deletion mutants are far more sensitive to Ab-mediated neutralization, suggesting that HVR1 also acts to conceal epitopes sensitive to neutralization (134–136). nAb selection drives HVR1 sequence evolution in chronically infected patients, while HVR1 remains stable over time in immunoglobulin-deficient patients (137–139). While HVR1 was predicted to be close to the CD81-binding site, Kong and colleagues have suggested that HVR1 lies on the opposite side of E2 molecule, where it masks a hydrophobic surface that is very sensitive to nAbs (45).

E1 and E2 are heavily glycosylated, particularly the immunodominant E2 (Figure 2C). E2 contains up to 11 N-linked glycosylation sites, most of them highly conserved across the different genotypes [reviewed in Ref. (140)]. The N-linked glycans of the ectodomains of E1E2 are reported to contribute almost 50% to the apparent molecular weight of these proteins, and are thought to limit nAbs’ access to key neutralization epitopes (35, 141). These glycans are also essential for the structure and function of E1E2, and play critical roles in viral entry (37, 142–144). Removal of the glycan shield increases the sensitivity of HCVpp to nAb activity (143).

The HCV particle is closely associated with lipoproteins (145), and this association reduces HCV’s buoyant density. Low-density and very low-density virions are more infectious than high-density particles (146, 147). The neutralization of HCVcc by nAbs increased with virion density, suggesting that lipoproteins masked neutralizing epitopes (148). Significantly more host-derived apoE was incorporated into HCV virions than E2, making it far more difficult for the humoral immune system to target E2 (82). Interestingly, apoC-I, the major structural protein of high-density lipoproteins (HDL), is also incorporated in virions (82, 149, 150). SR-BI binds HDL and is a known HCV entry factor, suggesting that HCV has evolved to exploit the normal HDL – SR-BI interaction to avoid the humoral immune system and expedite the virus lifecycle (151). Lipoproteins aid HCV evasion of humoral immunity through two mechanisms: first, the close association of HCV with low-density and very low-density lipoproteins cloaks the virus, thus protecting it from nAbs (Figure 2D) (82, 152) and secondly, HDL expedites virus entry (153).

*In vitro* and *in vivo* data suggest that HCV can spread by cell-to-cell transmission (22, 154, 155). Such cell-to-cell spread may enable HCV to bypass extracellular fluids, thereby denying nAbs access to viral particles; indeed, this mechanism appears to be resistant to Ab-mediated neutralization (Figure 2E) (156). CD81, claudin-1, occludin, and SR-BI have pivotal roles in the lateral transmission of HCV, although virions that were not dependent on SR-BI for transmission were significantly more sensitive to nAbs (156, 157). The importance of SR-BI in cell-to-cell spread of HCV would suggest that targeting SR-BI might be valuable for preventing cell-to-cell transmission and avoidance of the humoral immune response. It is, therefore, critical we elucidate the mechanism of cell-to-cell transmission.

Competition between interfering Abs and nAbs can disrupt virus neutralization by nAbs (Figure 2F). Interfering Abs are proposed to work in two ways: first, by directly competing with nAbs for the same epitope; second, by binding an epitope near a neutralizing epitope, thereby masking it. The role of interfering Abs in HCV is controversial (158–163). Abs binding E2 residues 436–447 interfere with nAbs binding to a CD81-binding domain containing E2 residues 412–421 (58). In contrast, a second study failed to find any interfering activity and reported the neutralizing activity was augmented by Abs binding both the 436–447 region and 412–421 region concurrently (162). Further study is needed to tease apart the mechanisms of interference, especially when considering using monoclonal Abs in passive immunizations and vaccine design.

Ideally, an effective HCV vaccine will need to generate a broad and highly reactive immune response at the first signs of HCV infection, before the virus has the chance to unleash its many immune escape mechanisms. A vaccine would need to target multiple antigenic determinants, thus raising the genetic barrier for mutational escape.

## Looking Forward

The new DAAs will dramatically improve HCV outcome. However, the development of a prophylactic or therapeutic vaccine is needed to control the global HCV problem. Successful vaccine development is dependent on our understanding of the immune response to HCV infection. In particular, it is imperative that we understand why some patients clear the virus naturally and how they are protected from reinfection. The relativity low cost and high benefit of vaccines have made them the cornerstone of modern public health strategies. To date, all successful viral vaccines elicit nAbs (123). The burning question of whether the humoral immune system can mediate or contribute to the clearance of HCV is still unanswered. In the majority of HCV infections, slow development of an nAb response allows HCV to establish widespread and persistent infection. For the lucky minority, the humoral immune system mounts a rapid, broad attack on HCV, contributing to spontaneous clearance of infection. Perhaps the question is far too simple. Our immune system by its very nature is a multifaceted entity, where no one part acts in isolation from the whole. Would the question of the role of B-cells in HCV be best addressed by taking a systems approach to the problem? Is it the failure of CD4^+^ T helper cells that ultimately leads to the failure of the humoral immune response, and thus the failure to control the infection? Do the other immune cells remain silent or do they engage with B-cells? As we enter the big data era in science, we may be better positioned to answer some of these questions.

## Conflict of Interest Statement

The authors declare that the research was conducted in the absence of any commercial or financial relationships that could be construed as a potential conflict of interest.
